# Direct visualization of interstitial flow distribution in aortic walls

**DOI:** 10.1038/s41598-022-09304-8

**Published:** 2022-03-30

**Authors:** Wataru Fukui, Yoshihiro Ujihara, Masanori Nakamura, Shukei Sugita

**Affiliations:** 1 Department of Electrical and Mechanical Engineering, Graduate School of Engineering, Nagoya Institute of Technology, Gokiso-cho, Showa-ku, Nagoya, 466-8555 Japan; 2 Center of Biomedical Physics and Information Technology, Nagoya Institute of Technology, Nagoya, Japan; 3 Department of Nanopharmaceutical Sciences, Nagoya Institute of Technology, Nagoya, Japan

**Keywords:** Biophysical methods, Optical imaging, Cardiovascular biology

## Abstract

Vascular smooth muscle cells are exposed to interstitial flow across aortic walls. Fluid shear stress changes the phenotype of smooth muscle cells to the synthetic type; hence, the fast interstitial flow might be related to aortic diseases. In this study, we propose a novel method to directly measure the interstitial flow velocity from the spatiotemporal changes in the concentration of a fluorescent dye. The lumen of a mouse thoracic aorta was filled with a fluorescent dye and pressurized in ex vivo. The flow of the fluorescent dye from the intimal to the adventitial sides was successfully visualized under a two-photon microscope. The flow velocity was determined by applying a one-dimensional advection–diffusion equation to the kymograph obtained from a series of fluorescent images. The results confirmed a higher interstitial flow velocity in the aortic walls under higher intraluminal pressure. A comparison of the interstitial flow velocity in the radial direction showed faster flow on the more intimal side, where hyperplasia is often found in hypertension. These results indicate that the proposed method can be used to visualize the interstitial flow directly and thus, determine the local interstitial flow velocity.

## Introduction

The pressure difference between the lumen and the outside of the aorta causes an interstitial flow across the aortic walls: fluid in the lumen enters the aortic walls^1^ and moves toward the adventitial side. Previous studies revealed an average flow velocity of ~ 0.05 μm/s^1–6^. This fluid flow provides mechanical stresses to the smooth muscle cells (SMCs) in the aortic walls, that is, it exposes SMCs to fluid shear stress. Computer simulations estimated the magnitude of the shear stress on SMCs as ~ 1 Pa^7,8^. This value is not negligibly small compared to the shear stress on the endothelial cells.

In vitro studies have reported that SMCs respond to shear stress. For example, SMCs incubated on a culture dish and subjected to shear stress increase the production rate of nitric oxide and endothelial nitric oxide synthase^9^, prostaglandin I_2_ and E_2_^10^, and platelet-derived growth factor and basic fibroblast growth factor^11^. SMCs embedded in collagen gels and subjected to shear stress also produce prostaglandin I_2_ and E_2_^10^. Interestingly, SMCs subjected to shear stress reduce the expression of contractile marker proteins such as smooth muscle myosin heavy chain, smoothelin, and calponin, indicating that an interstitial flow changes SMCs from a more contractile to a more synthetic state^12^. The synthetic phenotype of SMCs is often seen in aortic diseases such as atherosclerosis^13^, hypertension^14^, and aneurysms^15^. These indicate that the high shear stress due to the interstitial flow might be related to aortic diseases.

Previous studies measured the average interstitial flow velocity in the aorta by measuring the liquid volume leaking out from the outer surface of the aorta and dividing it by the outer surface area^2–5,16–23^. However, the surface area used for the calculation of the velocity includes extracellular matrix and cells spaces, where the liquid does not pass as flow. Thus, the averaged velocity values do not express the true velocity of the fluid. Furthermore, the interstitial flow velocity may differ at different locations in the aortic walls because the aortic walls are heterogeneous. For example, collagen fibers are abundant on the dorsal and proximal sides relative to the ventral and distal sides^24,25^, and space in the dorsal side is smaller than that in the ventral side^26^. Furthermore, smooth muscle-rich layers (SMLs) and elastic laminas (ELs), whose constituents are completely different, alternate in the radial direction. Because aortic diseases such as atherosclerosis and aneurysms were locally seen in the aorta, a method to determine the flow velocity distribution in the aorta is required.

In this study, we propose a novel method to quantify the local interstitial flow velocity. In this method, a fluorescent dye is allowed to flow in the aortic walls, and the flow velocity is determined from changes in the intensity of the fluorescent dye using a one-dimensional advection–diffusion equation. Since the direction of the interstitial flow is mainly radial, and the aortic tissue is much more heterogeneous in the radial direction than in other directions, we used the one–dimensional (instead of the two- or three-dimensional) advection–diffusion equation. This method is applied to measure the flow velocity distribution in the radial direction.

## Methods

### Sample preparation

Five Slc:ddY mice (8–11 weeks, 30–42 g, Chubu Kagaku Shizai, Nagoya, Japan) were used as a test model. All animal experiments were approved by the Institutional Review Board of Animal Care at Nagoya Institute of Technology, following recommendations from their Guide for Animal Experimentation and this study is reported under ARRIVE guidelines (https://arriveguidelines.org). The thoracic aorta was excised as reported in previous studies^27,28^. After the mouse was euthanized in a CO_2_ chamber, its thoracic aorta was exposed. As a length marker in the longitudinal direction in vivo, gentian violet dots were marked at 3-mm intervals on the surface of the aorta. After intercostal arteries were cauterized, the aorta was resected. The tubular sample was immersed in a buffer (0.5 mM CaCl_2_, 23.1 mM NaCl, 0.9 mM KCl, 0.2 mM MgSO_4_, 4.4 mM NaHCO_3_, 0.2 mM KH_2_PO_4_, 1.8 mM glucose) until the next experiment to maintain the activity of the SMCs in the aorta.

### Intraluminal pressurization

**Figure 1:**
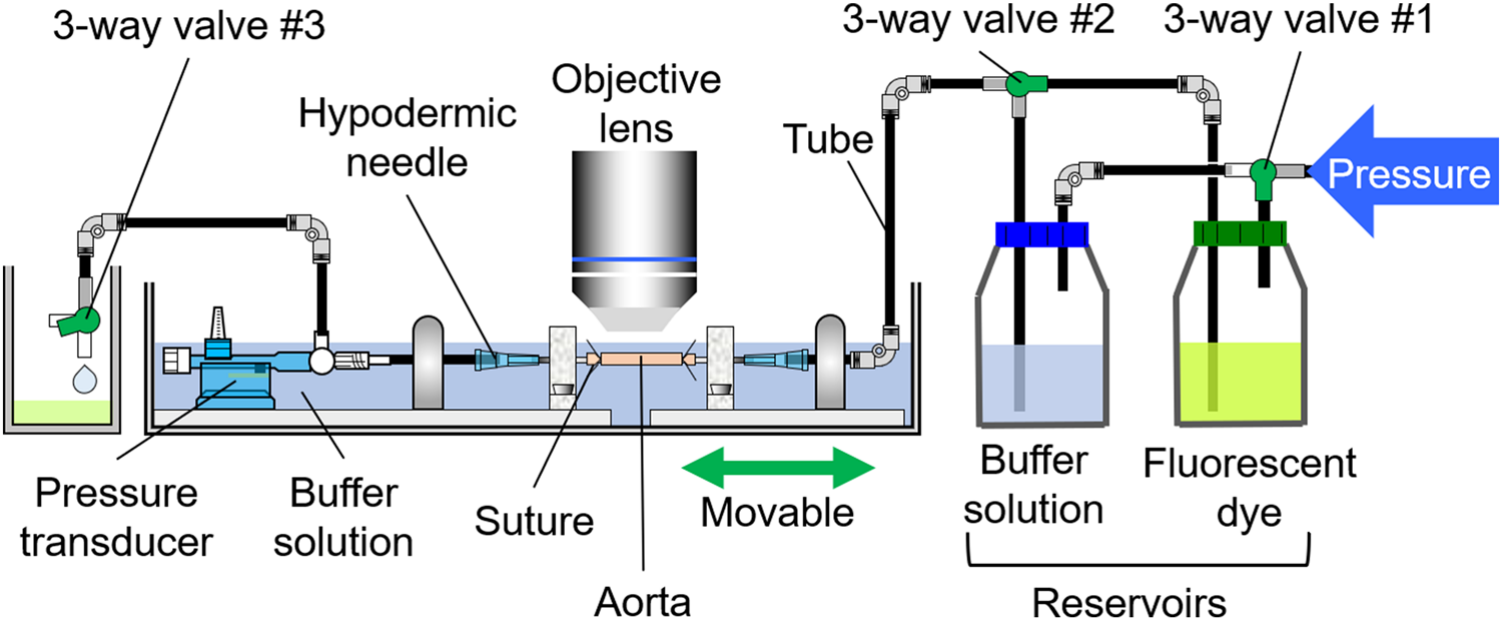
Schematic of experimental device for applying intraluminal pressure to a vessel. This device is a slightly modified version of the one used in Sugita et al.^28^ The image was created using the Microsoft PowerPoint for Microsoft 365 MSO (16.0.14326.20782).

The obtained tubular sample was pressurized in a manner similar to that in previous studies^27,28^. Figure 1 shows a schematic of the experimental setup. The air pressure was regulated using an electropneumatic regulator (640BA20B, Asahi Enterprise, Tokyo, Japan) connected to a pressure source (0.3 MPa), a digital-analogue/analogue–digital (DA/AD) converter (NI USB-6363, National Instruments, Austin, TX, USA), a personal computer (FMV BIBLO, Fujitsu, Tokyo, Japan; PC), and NI LabVIEW 2010 software (National Instruments). The pressure was applied to two reservoirs to convert air pressure to liquid pressure: one reservoir was filled with the buffer, and the other one was filled with 1.06 mM of uranine fluorescent dye solution (CI-45350, Tokyo Chemical Industry, Tokyo, Japan). An aortic sample was placed downstream of the reservoirs. Both ends of the aortic sample were tied to hypodermic needles (NN-2332R, Terumo, Tokyo, Japan) with suture threads (C-23-N2 7-0, Natsume Seisakusho, Tokyo, Japan), and the needles were fixed on a tissue bath. The sample was stretched in its longitudinal direction until the intervals of the length marker reached 3 mm. A pressure transducer (DX-300, Nihon Kohden, Tokyo, Japan) at the downstream side of the sample measured the pressure inside the tube through a strain amplifier (DPM-911B, Kyowa Electronic Instruments, Chofu, Japan), the DA/AD converter, and the software installed on the PC. The downstream side of the tube was connected to an outlet bath through a three-way valve (no. 3 in Fig. 1).

### Fluorescent microscopy

Fluorescent light was observed under a two-photon microscope (FV1200MPE, Olympus, Tokyo, Japan). A Ti:sapphire laser (wavelength: 800 nm, strength: 2.0%) was applied to the sample through a 60 × objective lens (LUMPLFLN60XW, Olympus) and a V/G filter (FV-10-MRV/G, Olympus). To specify the position of the aorta, autofluorescence from the elastin in the aorta was mainly observed in a V-channel bandpass filter (420–460 nm). The fluorescent light of the fluorescent dye solution was imaged using a G-channel bandpass filter (495–540 nm).

### Experimental protocol

The intraluminal pressure *P* was set at 40 mmHg, and only the buffer was introduced into the aorta by switching three-way valves nos. 1 and 2 (Fig. 1). The flow rate was controlled such that the difference between the set and the measured pressures did not exceed 10 mmHg with three-way valve no. 3. The ELs were imaged in the plane perpendicular to the radial (*r*) direction, with an image size of 1 pixel (0.41 μm) in the longitudinal (*z*) and 512 pixels (211.97 μm) in the circumferential (*θ*) directions. By moving the objective lens in the *r*-direction in steps of 0.41 μm, the *r*–*θ* cross-sectional image was obtained.

The fluorescent dye solution was introduced into the aorta by switching three-way valves nos. 1 and 2. The aorta was imaged as stated above as a time-lapsed image with an interval of 2.7–3.5 s. Image capture was continued for 3–5 min. After imaging, the buffer solution was reintroduced into the aorta to wash out the fluorescent solution. The new position in the sample was then selected, and intraluminal pressurization and image capture were similarly repeated at 80, 120, and 160 mmHg. All experiments were completed within 24 h after the resection of the sample.

### Image analysis

**Figure 2:**
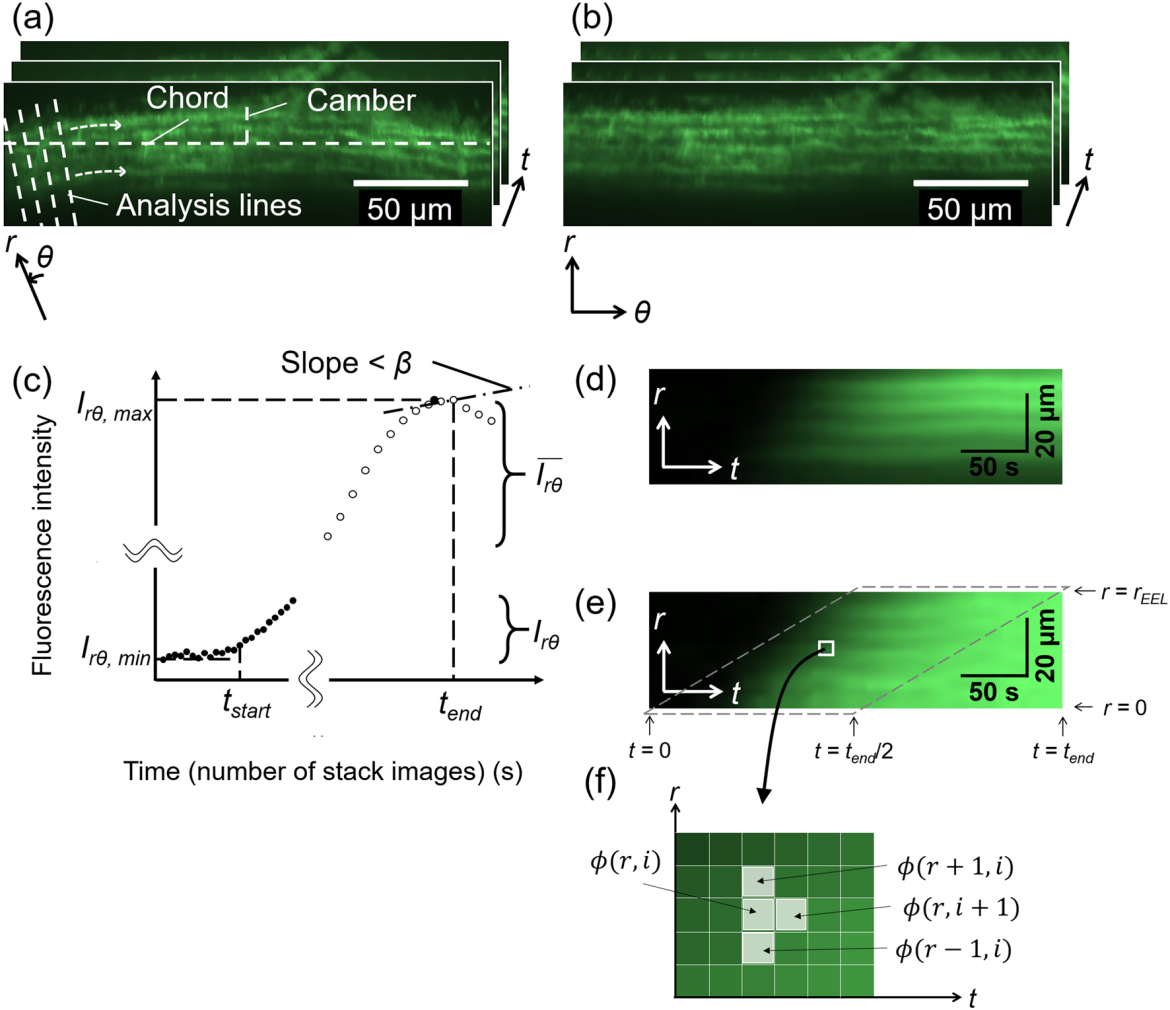
Schematic of image analysis. (**a**) An image stack of fluorescent dye captured in the radial-circumferential (*r*–*θ*) plane. (**b**) To convert the image of the aortic wall on an arch to images of a flattened one, the chord and camber lengths were measured from any arbitrary EL (outermost layer in this figure) and the center coordinates of the arc were determined. An analysis line was drawn from the center coordinates to an arbitrary position on the left edge of the images and rotated clockwise around the center coordinates. The fluorescence intensity on each line was recorded, and the image stack was reproduced from the intensity data. (**c**) Identification of start time *t*_*start*_ and end time *t*_*end*_. The average fluorescence intensity $I_{r\theta } \left( t \right)$ in the aortic region of each slice was measured (closed circles), and the minimum $I_{r\theta ,min}$ and maximum $I_{r\theta ,max}$ were measured. *t*_*start*_ was defined as the time *t* when $I_{r\theta } \left( t \right)$ slightly exceeded $I_{r\theta ,min}$ (see main text for precise definition). The moving average of the fluorescence intensity $\overline{{I_{r\theta } }} \left( t \right)$ (open circles) was calculated, and *t*_*end*_ was defined as the time *t* when $\left\{ {\overline{{I_{r\theta } }} \left( t \right) - \overline{{I_{r\theta } }} \left( {t - 1} \right)} \right\}$ first became less than *β*. (**d**) A kymograph obtained by reslicing image (**b**) and averaging it on the *θ*-axis. (**e**) A normalized kymograph with 0 at *t*_*start*_ and 1 at *t*_*end*_. Normalization was performed at each *r*-coordinate. Further image analysis was performed in the region bordered by the broken line. (**f**) Magnified view of the kymograph. The differential of the intensity was calculated for each pixel. These images were taken at 80 mmHg. The graph was created using the Microsoft Excel for Microsoft 365 MSO (16.0.14326.20782). The image was created using ImageJ 1.52a.

Image analysis was performed using image analysis software (ImageJ 1.52a, National Institutes of Health, Bethesda, MD, USA). Figure 2 shows a schematic of the image analysis process. The drift of the time-lapsed image stack obtained as described above was compensated by performing image correlation. The chord length and camber of the arch of any EL were measured, a circle was fitted to the EL, and its center coordinates were determined (Fig. 2a). By regarding the center coordinates as the origin of the polar coordinates, the image stack with polar coordinates was converted to an image stack with Cartesian coordinates (Fig. 2b). The converted radial direction still has an image resolution of 0.41 μm/pixel. The image stack was converted to time-lapsed images with 1 s intervals by linearly compensating every two sequential images.

There was a time delay until the fluorescent solution reached the sample position from the reservoir after the valves were opened. Thus, the start time *t*_*start*_ of the analysis was determined from the fluorescent intensity changes (Fig. 2c). First, the average intensity $I_{r\theta } \left( t \right)$ of the image in the aortic region was calculated at each slice (*i.e.*, each time *t*), and its maximum $I_{r\theta ,max}$ and minimum $I_{r\theta ,min}$ were determined. Then, the time *t* when $\left\{ {I_{r\theta } \left( t \right) - I_{r\theta ,min} } \right\}$ first exceeded *α* times the difference between $I_{r\theta ,max}$ and $I_{r\theta ,min}$, that is,

1 $$I_{r\theta } \left( t \right) - I_{r\theta ,min} \ge \left( {I_{r\theta ,max} - I_{r\theta ,min} } \right) \times \alpha$$

was determined as *t*_*start*_. Then, the moving average $\overline{{I_{r\theta } }} \left( t \right)$ was calculated for time duration $t_{MA}$ as

2 $$\overline{{I_{r\theta } }} \left( t \right) = \mathop \sum \limits_{{t^{\prime} = t - t_{MA} }}^{t} \frac{{I_{r\theta } \left( {t^{\prime}} \right)}}{{t_{MA} }}.$$

The end time *t*_*end*_ of the analysis was determined as the time when changes in $\overline{{I_{r\theta } }} \left( t \right)$ first became less than *β* after *t*_*start*_ (Fig. 2c). In this study, *α* = 0.5%, $t_{MA}$ = 10 s, and *β* = 1 were selected. In the following analysis, only the time-lapsed images between *t*_*start*_ and *t*_*end*_ were used. The *r*–*θ* plane image stack was resliced into the *r*–*t* plane image stack to obtain the so-called kymograph stack, and this stack was then averaged in the *θ* direction (Fig. 2d). Images of only the media, from the internal to the external ELs, were cropped, and the image size was reduced to one-fifth the original for averaging the local intensities. The intensity $I_{rt} \left( {r,{ }t_{start} } \right)$ was subtracted from the intensity $I_{rt} \left( {r,{ }t} \right)$ at the coordinates $\left( {r,{ }t} \right)$ in the kymograph to remove the background value as follows:

3 $$I^{\prime}_{rt} \left( {r,{ }t} \right) = I_{rt} \left( {r,{ }t} \right) - I_{rt} \left( {r,{ }t_{start} } \right).$$

Then, the obtained intensity $I^{\prime}_{rt} \left( {r,{ }t} \right)$ was normalized by the intensity $I^{\prime}_{rt} \left( {r,{ }t_{end} } \right)$ at *t*_*end*_ as follows (see Fig. 2e):

4 $$I^{\prime\prime}_{rt} \left( {r,{ }t} \right) = I^{\prime}_{rt} \left( {r,{ }t} \right)/I^{\prime}_{rt} \left( {r,t_{end} } \right).$$

This process was performed to eliminate the effect of the difference in the intensity in the radial direction because the light at deeper tissues (intimal side) tends to be weak and dispersed owing to the long distance from the objective lens. The obtained intensity $I^{\prime\prime}_{rt} \left( {r,{ }t} \right)$ in the *r*–*t* plane was used in the following analysis.

### Calculation of velocity and diffusion coefficient

To measure the interstitial flow in the *r*-axis, the following one-dimensional advection–diffusion equation was used:

5 $$\frac{\partial \phi }{{\partial t}} + v\frac{\partial \phi }{{\partial r}} - k\frac{{\partial^{2} \phi }}{{\partial r^{2} }} = 0.$$

here $\phi$ is the concentration of the fluorescent solution; *v*, the interstitial flow velocity; and *k*, the diffusion coefficient. The discretization of Eq. (5) gives

6 $$\frac{{\phi \left( {r, t + 1} \right) - \phi \left( {r, t} \right)}}{\Delta t} + v\frac{{\phi \left( {r + 1, t} \right) - \phi \left( {r, t} \right)}}{\Delta r} - k\frac{{\phi \left( {r + 1, t} \right) - 2\phi \left( {r, t} \right) + \phi \left( {r - 1, t} \right)}}{{\left( {\Delta r} \right)^{2} }} = 0,$$

where $\phi \left( {r, t} \right)$ is the concentration at a coordinate $\left( {r, t} \right)$ in the kymograph (see Fig. 2f). The coefficient of each term in Eq. (6) is expressed as

7 $$a = \frac{{\phi \left( {r, t + 1} \right) - \phi \left( {r, t} \right)}}{\Delta t}, b = \frac{{\phi \left( {r + 1, t} \right) - \phi \left( {r, t} \right)}}{\Delta r}, c = \frac{{\phi \left( {r + 1, t} \right) - 2\phi \left( {r, t} \right) + \phi \left( {r - 1, t} \right)}}{{\left( {\Delta r} \right)^{2} }}.$$

Equation (6) is then expressed as

8 $$a^{\prime} = vb^{\prime} + k,$$

where

9 $$a^{\prime } = \frac{a}{c},b^{\prime } = - \frac{b}{c}$$

In the preliminary experiment to determine the relationship between the concentration and intensity (Fig. S1.1), we obtained a strong and linear correlation between them (Fig. S1.2, see Supplementary Material S1). Thus, the intensity in the normalized kymograph shown in Fig. 2e was considered to reflect the concentration of the fluorescent dye:

10 $$\phi \left( {r, t} \right) = I^{\prime\prime}_{rt} \left( {r,{ }t} \right).$$

Further, the application of Eq. (6) to each pixel in the kymograph produced many combinations of (*a′*, *b′*). These combinations were plotted on a graph with ordinate *a′* and abscissa *b′*. To eliminate the small intensity changes that often produce extremely high values in the calculation of the differentiation, the kymograph in the ranges with

11 $$\frac{{2r_{EEL} }}{{t_{end} }}t - r_{EEL} \le r \le \frac{{2r_{EEL} }}{{t_{end} }}t\, for\, 0 \le r \le r_{EEL}$$

were used for further analysis, where $r_{EEL}$ is the radial coordinate at the external elastic lamina (EEL, Fig. 2e). The extreme data (*a′*, *b′*) were excluded using the modified Stahel–Donoho method (Wada, 2010) in R software (v3.5.1, University of Auckland, Auckland, North Island, NZ). A linear line was fitted to the plots (*a’*, *b’*) with a least-squares regression, and the slope and *b’*-axis intercept were determined as *v* and *k*, respectively, using Eq. (8).

### Distribution of interstitial flow velocity in radial direction

The distribution of the interstitial flow velocity in the radial direction was measured. In the kymograph image, a local area with a length of 5–10 pixels (ca. 2–4 µm) in the radial direction was selected in the EL and SML regions for the autofluorescence image of elastin. From the intimal side, these ELs were named EL1, EL2, and EL3, and the SMLs were named SML1, SML2, and SML3. Then, the abovementioned process was applied to the image to obtain *v*, except that the image size was reduced to one-fifth the original for averaging the local intensities.

To determine the radial position of the local area image, the normalized distance *d* from the internal elastic lamina (IEL) was calculated as the distance from the IEL divided by the distance between the IEL and the EEL.

### Statistical method

The correlation coefficients *R* between plots such as *a′*–*b′*, *v*–*P*, *k*–*P*, and *v*–*d* were tested using Student’s *t*-test. The velocity difference between SMLs was tested using the Steel–Dwass test. Data were expressed as mean ± standard deviation (SD). A significance level of *p* = 0.05 was used.

## Results

### Observation of fluorescent solutions during intraluminal pressurization

**Figure 3:**
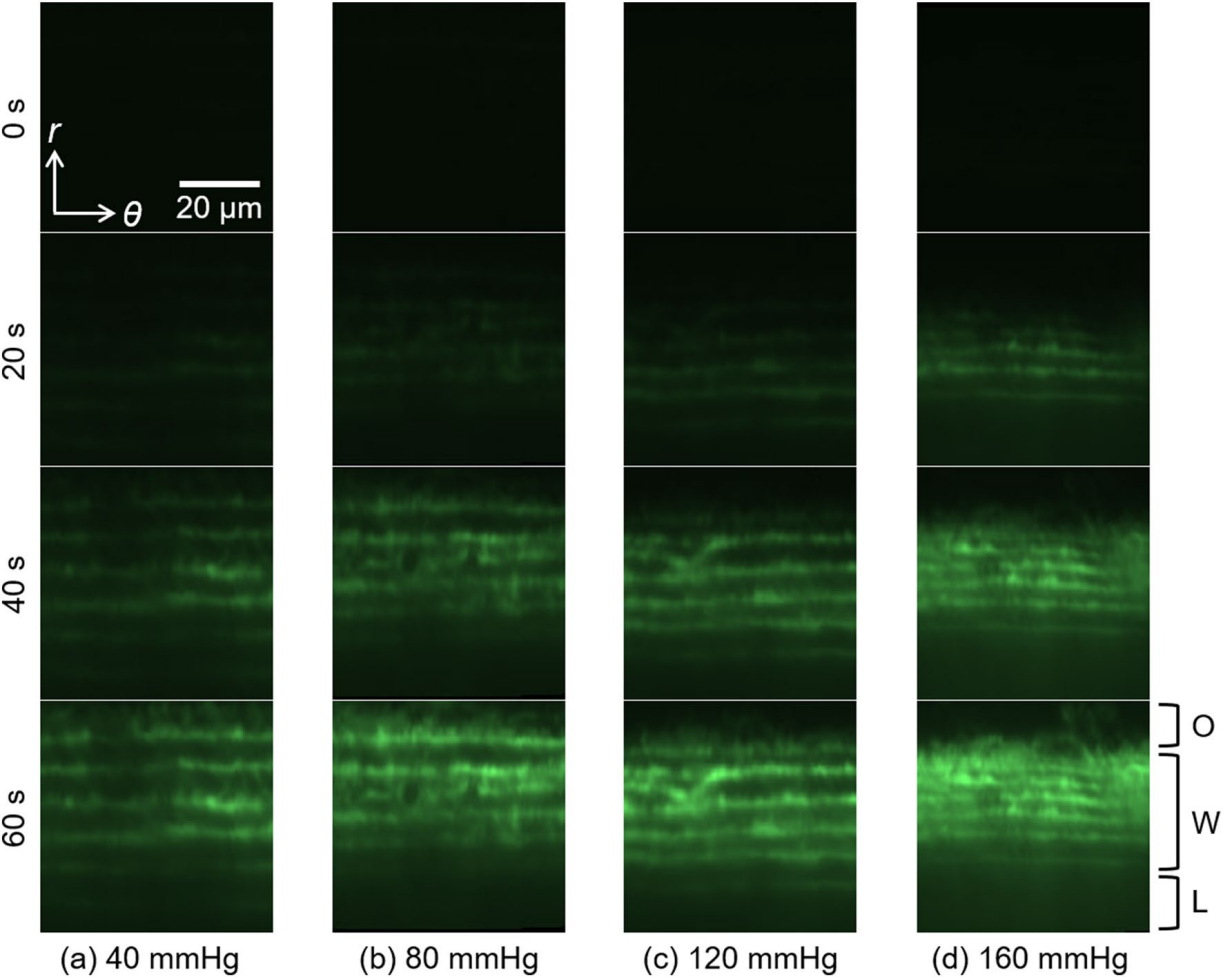
Time-lapsed images of fluorescence in the aortic walls in the radial-circumferential (*r*–*θ*) cross-section at intraluminal pressures of (**a**) 40, (**b**) 80, (**c**) 120, and (**d**) 160 mmHg at 0, 20, 40, and 60 s after *t*_start_. From the top to the bottom sides, the outside of the aorta (O), aortic wall (W), and lumen (L) are shown. The image was created using ImageJ 1.52a.

Figure 3 and Movie 1 show raw time-lapsed images of the fluorescent solution in the thoracic aorta in the *r*–*θ* plane under intraluminal pressurization. After the fluorescent solution reached the aorta from the reservoir, a high-intensity area spread from the bottom (*i.e.*, intimal side) to the top (*i.e.*, adventitial side). The high intensity reached the adventitial side more quickly under a higher intraluminal pressure. Although this is the fluorescent dye channel image and not the main elastin autofluorescence channel, the EL was clearly observed after the fluorescent dye reached the aortic walls.

**Figure 4:**
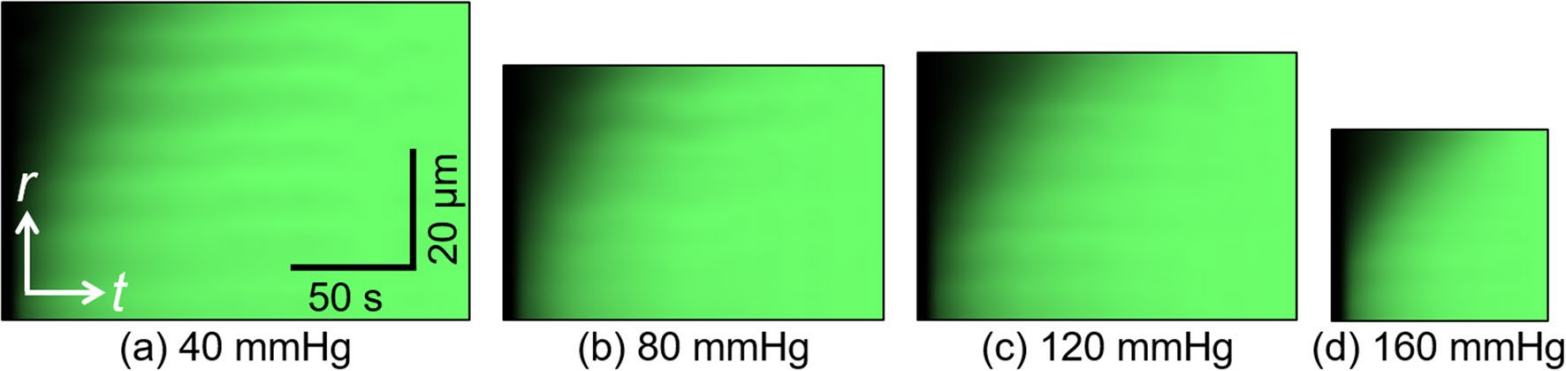
Kymograph of fluorescent dye solution at intraluminal pressures of (**a**) 40, (**b**) 80, (**c**) 120, and (**d**) 160 mmHg. *t*, time axis; *r*, radial axis. These images were obtained from the images shown in Fig. 3. The image was created using ImageJ 1.52a.

Figure 4 shows the kymographs at 40, 80, 120, and 160 mmHg. These kymographs show the movement of the higher intensity from the intimal to the adventitial side, indicating that the fluorescent dye flows through the aortic walls. Furthermore, the time length of the kymograph tended to be shorter at higher intraluminal pressures. The end time of the kymograph was 222 ± 75 s at 40 mmHg, 211 ± 69 s at 80 mmHg, 166 ± 53 s at 120 mmHg, and 127 ± 6 s at 160 mmHg. The end time is negatively and significantly correlated with the pressure. This result indicates that the fluorescent intensity at higher pressure reached a stable state more quickly. In this kymograph, stripe patterns were also seen under all pressure conditions. The higher-intensity regions in the strip patterns correspond to the region of ELs.

### Interstitial flow velocity and diffusion coefficient in analysis of whole thickness

**Figure 5:**
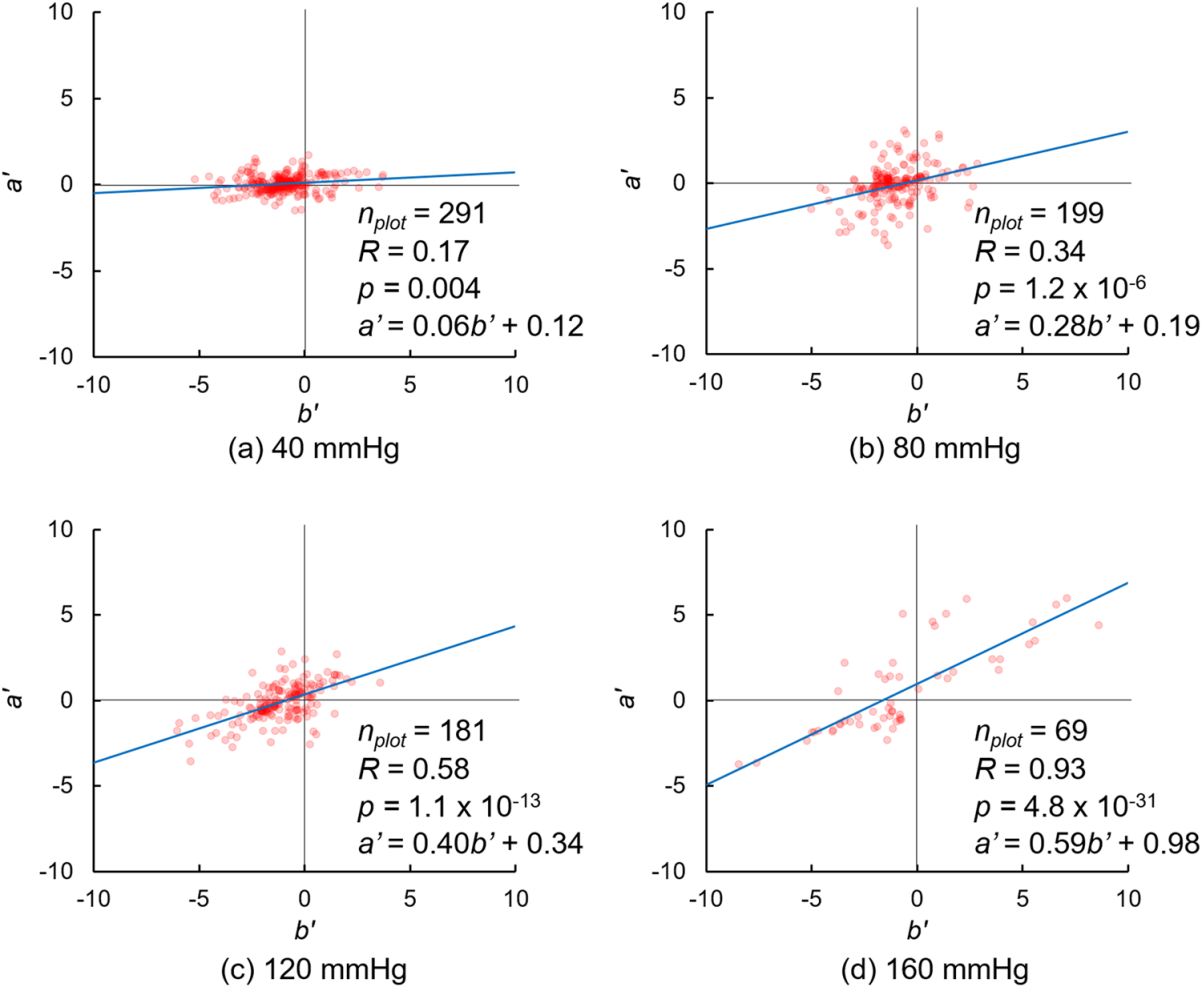
Plots of *a’* and *b’* as given by Eq. (8) at intraluminal pressures of (**a**) 40, (**b**) 80, (**c**) 120, and (**d**) 160 mmHg. *n*_*plot*_, number of plots. These graphs are obtained from the images shown in Figs. 3 and 4. The correlation coefficient *R* was tested using the Student’s *t*-test. The graph was created using the Microsoft Excel for Microsoft 365 MSO (16.0.14326.20782).

Figure 5 shows typical graphs of combinations of (*a′*, *b′*). The density of the plots was high from the bottom left to the top right, indicating that the velocity expressed as the slope of the fitted line is positive. The slope of the fitting line increased with increasing pressure, as given by *a′* = 0.06*b′* + 0.12 at 40 mmHg (Fig. 5a), *a’* = 0.28*b′* + 0.19 at 80 mmHg (Fig. 5b), *a′* = 0.40*b′* + 0.34 at 120 mmHg (Fig. 5c), and *a′* = 0.59*b′* + 0.98 at 160 mmHg (Fig. 5d). The number of plots tended to decrease at higher intraluminal pressures because the image sizes of the kymograph under such conditions were small (Fig. 4).

**Figure 6:**
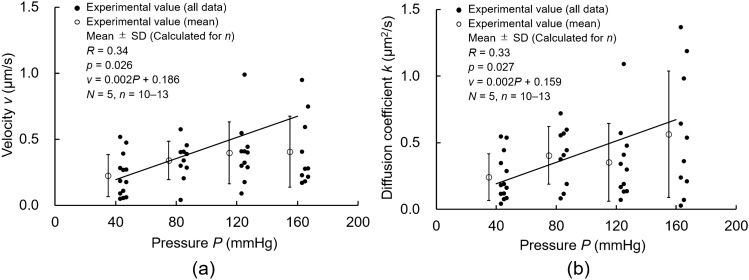
Correlations between intraluminal pressure *P* and both (**a**) interstitial flow velocity *v* and (**b**) diffusion coefficient *k*. *N*, number of mice; *n*, number of data*; R*, correlation coefficient. The correlation coefficient *R* was tested using the Student’s *t*-test. The graph was created using the Microsoft Excel for Microsoft 365 MSO (16.0.14326.20782).

Figure 6 shows plots of the interstitial flow velocity *v* and diffusion coefficient *k* against the pressure *P*. Because a significant correlation between *a′* and *b′* was not obtained from data at 80 mmHg and the adventitial side was destroyed owing to the laser as indicated by the data at 160 mmHg, these data were not used in the following analysis. The velocities *v* increased with increasing pressure *P*; 0.23 ± 0.16 μm/s at 40 mmHg, 0.34 ± 0.15 μm/s at 80 mmHg, 0.40 ± 0.23 μm/s at 120 mmHg, and 0.40 ± 0.27 μm/s at 160 mmHg. Further, the diffusion coefficients *k* increased with increasing pressure *P*; 0.24 ± 0.18 μm^2^/s at 40 mmHg, 0.41 ± 0.22 μm^2^/s at 80 mmHg, 0.35 ± 0.29 μm^2^/s at 120 mmHg, and 0.56 ± 0.47 μm^2^/s at 160 mmHg. The correlation coefficients for the *P*–*v* and *P*–*k* relationships were both significant.

### Velocity distribution in radial direction

**Figure 7:**
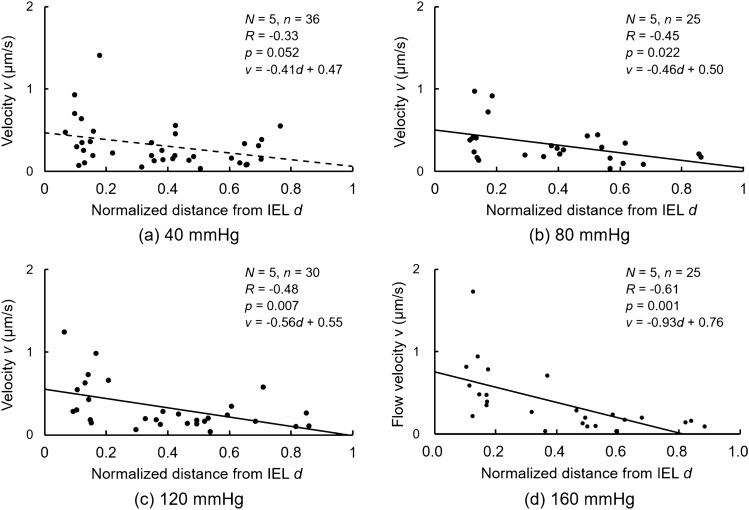
Correlations between normalized distance *d* from the IEL and interstitial flow velocity *v* at intraluminal pressure *P* of (**a**) 40, (**b**) 80, (**c**) 120, and (**d**) 160 mmHg. *N*, number of mice; *n*, number of data*; R*, correlation coefficient. The correlation coefficient *R* was tested using the Student’s *t*-test. The graph was created using the Microsoft Excel for Microsoft 365 MSO (16.0.14326.20782).

**Figure 8:**
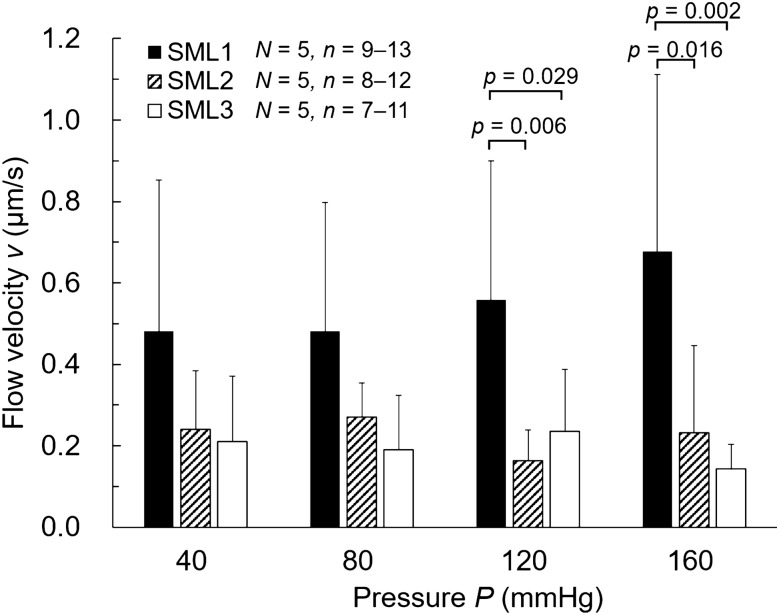
Comparison of flow velocity *v* between SML1–SML3. *N*, Number of mice; *n*, Number of data. Data were tested using the Steel–Dwass test. The graph was created using the Microsoft Excel for Microsoft 365 MSO (16.0.14326.20782).

Figure S2.1 shows the kymograph in the local EL and SML regions. The intensity change in the radial direction was negligible, whereas that in the time direction was recognizable. Figure S2.2 shows the plots of *a′* and *b′* in the EL and SML regions. Significant correlations were obtained, as seen in the analysis for the whole wall thickness (Fig. 5). The slope of the relationship between *a′* and *b′* was steeper in the SML region and intimal side. Figure S2.3 shows the slope, that is, the interstitial flow velocity *v*. In ELs, these velocities were lower or sometimes negative. Thus, the reliability of the velocity in ELs was not sufficient, and only the SML data were used. Figure 7 shows the interstitial flow velocity *v* in the SMLs plotted to the normalized radial position *d* for all pressure levels. Negative correlations were observed at 40–160 mmHg, and the correlation coefficient was significant at 80–160 mmHg. Figure 8 shows the interstitial flow velocity *v* in SML1–SML3. At 120 and 160 mmHg, *v* in SML1 was significantly higher than that in SML2 and SML3. At 40 and 80 mmHg, *v* in SML1 tended to be higher than that in other SMLs, and significant differences were not seen. These results indicate that the interstitial flow velocity is higher on the intimal side than on the adventitial side.

## Discussion

In this study, we proposed a novel method to quantify the local interstitial flow velocity in the aortic wall. Upon applying an intraluminal pressure to the mouse thoracic aorta, the fluorescent dye solution introduced in the lumen was observed to move from the intimal to the adventitial side under a two-photon microscope. This method directly observed the flow through the change in the concentration of the fluorescent dye. By applying a one-dimensional advection–diffusion equation to time-lapsed images of the fluorescence intensity, the flow velocity and diffusion coefficient were successfully obtained. The interstitial flow velocity in the radial direction was higher under higher intraluminal pressure as obtained in previous studies. Furthermore, this method reveals the much higher interstitial flow velocity in the intimal side of the aorta as estimated in only the simulation study^29^.

In this study, the interstitial flow velocity in the ex vivo mice aorta was respectively measured to be 0.34 and 0.40 µm/s at 80 and 120 mmHg on average; these values were higher than previously reported ones. The velocity in ex vivo aortas at 100 mmHg was reported as 0.055 µm/s^1^, 0.052 µm/s^2^, and 0.045 µm/s^3^ in rabbits and ~ 0.03 µm/s^4^, 0.025 µm/s^5^, and ~ 0.03 µm/s^6^ in rats. Previous studies reported the averaged interstitial velocities determined by dividing the flow rate by the area of the intraluminal surface. However, the actual area where liquid can flow in the aorta is smaller owing to the existence of the extracellular matrix and cells. Thus, the interstitial flow velocities measured in the previous studies underestimate the actual liquid flow velocity. Tedgui and Lever^26^ reported that the extracellular space was ~ 40% in the media at 70 mmHg. If the area in which the liquid can flow in the radial direction is 40% of the total area of the intraluminal surface, the actual flow velocity is 2.5 times the interstitial velocity measured in previous studies.

Although the abovementioned factor might have resulted in the higher interstitial velocity in our study, differences still exist between the interstitial flow in previous studies and our study. Various factors might cause these velocity differences. We investigated several possible factors that may affect interstitial velocities. The interstitial flow velocity with ECs is higher than that without ECs^3,4,6,21,30^, and the interstitial flow with the low viscosity solution is fast^16^. To investigate the effect of these factors, we measured the interstitial velocity in a region with ECs using viscosity regulated solution (See Supplementary Materials S3). However, the interstitial velocity was comparable to that given in Fig. 6, indicating that the difference in velocities between previous studies and our study was not caused by the EC detachment and difference in viscosity of the solution. The other possible factors are the use of different animals and the laser scanning. We also measured the interstitial velocity in the mouse aorta using the previous method. The preliminary result showed an interstitial velocity of ~ 0.04 μm/s, which was comparable to the previous velocity measured in aorta obtained from rabbits and rats. With regard to the effect of the laser, scanning using the two-photon microscope was performed from the intimal to the adventitial side at a speed of 25 µm/s in the radial direction. The interstitial flow velocity for all data in this study was 0.39 µm/s; this results in a 1.6% error in the interstitial flow velocity. The other factor for the higher interstitial velocity in our study is that, unlike in previous studies, the adventitia was removed to observe the fluorescence in the media (Fig. S4). Because the adventitia seems to resist the interstitial flow, its removal might result in the higher interstitial flow in this study.

The proposed method to measure the interstitial flow velocity differs from previous methods, in which the total volume of the liquid eluted from the aorta was measured and divided by the surface area, in several ways^2,3,5,16,18–20,22,23,31^. First, it can directly observe the interstitial flow in the aorta. Thus, the local difference in the interstitial flow can be investigated in the *z*-, *θ*-, and even *r*-directions, unlike in previous studies. Specifically, because the structure of ELs and SMLs is quite different, there might be a big difference between SMLs and ELs that might affect the local velocity in the *r*-direction. Second, it can measure the velocity for a limited time; by contrast, previous methods do not have a time limitation. Because the measurements using the proposed method depend on changes in the concentration of the fluorescent dye solutions, they cannot be performed after the concentration reaches a stable value.

This study directly and experimentally demonstrated higher interstitial flow velocity in the intimal side of the SML (Fig. 7). When the continuum theory is applied to the inner and outer surfaces, the interstitial flow velocity at the inner surface must be higher than that at the outer surface, because the outer surface area is larger owing to the cylindrical shape of the aorta. However, the maximum rate of decrease in the velocity was estimated to be only 8%. The curvature radius of the ELs at the intimal and adventitial sides was ~ 990 µm and ~ 1080 µm, respectively; in other words, the intimal surface area was 8% smaller than the adventitial surface area at 40 mmHg. The fitting lines in Fig. 7 indicate a much larger rate of decrease at the outer surface compared to that at the inner surface. Thus, other factors must influence the higher interstitial flow velocity in the more intimal side of the aorta. Through simulations, Huang et al.^29^ revealed much faster flow at the fenestral pores in the IEL, and Tada and Tarbell^7,32^ predicted that the flow throw the pores was jet flow. Thus, fenestral pores might influence the faster flow in the intimal side. The IEL is lost^33^ in the intracranial aneurysms. If the fenestration pores in the IEL cause faster interstitial flow in SML1, loss of IEL decreases the interstitial flow velocity in aortic tissues. Thus, the volume decrease of ELs might influence the shear stress on the smooth muscle cells, affecting the function of the cells.

Hypertensive rats showed aortic thickening, especially in the intimal side^34^; this might have been caused by the faster interstitial flow in the intimal side, as shown in this study. SMCs in collagen gel subjected to a pressure of 0.005 Pa reduced the production of contractile-type proteins, indicating that they changed into a synthetic phenotype^12^. Changes to the synthetic type improve the synthesis of collagen^35^. Because an increase in ground substances was also seen in hypertensive rats^34^, the higher shear stress induced by faster fluid flow might be a cause of the aortic thickening seen in hypertension. This issue requires further investigation.

The interstitial flow velocity *v* was obtained under several assumptions. First, we assumed a constant flow velocity during the experiment. Further, Lever et al.^18^ reported that the velocity decreased with time for ~ 10 min after intraluminal pressurization. Because the present study measures the velocity for a few minutes after pressurization, the interstitial flow velocity in the stable state might be lower than that observed in this study. The aorta is viscoelastic, and we have often observed its outward movement during pressurization. However, the aortic thickness remains almost constant (Fig. S5.1), and the effect of the viscoelasticity of the aorta should be negligibly small. Second, we considered that the fluorescent intensity reflected the concentration of the fluorescent dye solution. This is because the investigation of the relationship between the intensity and the concentration showed a significant and strong correlation (see Supplementary Materials S1), indicating that the intensity can be used as a concentration index.

An EL-like structure, namely, a stripe pattern, was observed in the fluorescent dye solution image (Fig. 3). Because a high intensity was not observed at the start time *t*_*start*_, this should not be the autofluorescence from elastin. When an aorta was sectioned perpendicular to the longitudinal direction and immersed in the fluorescent dye solution without pressurization, following which the dye was washed out with the buffer, a higher intensity was observed in ELs than in SMLs (see Supplementary Materials S6). Therefore, we assume that more fluorescent dye attaches to something in ELs rather than that in SMLs.

The flow velocity deviation was large at high pressure. One possible explanation for the large deviation at high pressure is the quick movement of the fluorescent dye. At high pressure, the fluorescent dye reaches the adventitial side faster, and the fluorescent intensity in the medial wall also reaches the plateau level faster. This results in the small size of the kymograph, as shown in the plots in Fig. 5. Since the interstitial velocity is obtained from the slope of the linear line fitting to the scatter plots, a small number of scatter plots might result in low reliability of the slope of the line. The intimal side also shows a large flow velocity deviation (Fig. 8). A possible reason is the image quality. In this study, we show radial-circumferential plane images, as shown in Fig. 3. However, these images were captured from the adventitial side and reconstructed to radial-circumferential plane images. The excitation and fluorescent lights should pass through more aortic walls and experience more scattering and noise. Thus, the image quality on the intimal side is poorer than that on the adventitial side. This image quality difference might result in large variability on the intimal side.

The proposed method can measure the diffusion coefficient *k* of the fluorescent dye in the aortic walls (Fig. 6b). Notably, even though both flow and diffusion conveyed the fluorescent dye from the lumen to the adventitial side, the flow, and not the diffusion, mainly conveyed fluorescent dyes because the Peclet number was much larger than 1 (see Supplementary Materials S7). Although the movement of the fluorescent dye by diffusion was much smaller than that by flow, the measured diffusion coefficient *k* seemed appropriate because the diffusion coefficient *k* under the condition without a flow showed a similar value to that under the condition with a flow (see Supplementary Materials S8). Interestingly, the diffusion coefficient increased with an increase in the intraluminal pressure. Tedgui and Lever^26^ reported that the porosity of the liquid to fill the media at 70 mmHg was lower than that at 180 mmHg. This indicates that voids in the media increased with increasing intraluminal pressure, resulting in a higher diffusion coefficient as obtained in this study.

There are some limitations to this study. One is that we measured the velocity distribution in only the radial direction. Although heterogeneity in the circumferential and longitudinal directions is lower than that in the radial direction, heterogeneous constituents do exist in the former two^24–26^. Moreover, we measured the institutional flow velocity in the relaxed state of the SMCs. Since the contraction/relaxation state of SMCs influences the mechanical properties of the aorta^36^, the flow velocity might change depending on the state of the SMCs.

## Conclusion

We proposed a novel method for directly observing the flow of a fluorescent dye and for quantifying the local interstitial flow velocity in the aortic wall. As a result, a higher interstitial flow in the radial direction was confirmed under the higher intraluminal pressurization of the aorta. Moreover, the interstitial flow was faster at the more intimal side of the aorta. These results indicate that the proposed method can be used to visualize the interstitial flow directly and thus, determine the local interstitial flow velocity.

## Supplementary Information

Supplementary Information 1.

Supplementary Video 1.

Supplementary Information 2.

## Data Availability

The datasets generated during and/or analyzed during the current study are available as a supplementary data file.
