# Supplementary material for: Direct visualization of interstitial flow distribution in aortic walls
Source: Sci Rep. 2022 Mar 30;12:5381. doi: 10.1038/s41598-022-09304-8 (PMC8969162; doi:10.1038/s41598-022-09304-8)
Supplement: Supplementary file 3 — Supplementary Information 2. [file 41598_2022_9304_MOESM3_ESM.pdf]

SUPPLEMENTAL MATERIAL

S1. Can the fluorescent intensity be used as an index of the concentration of fluorescent dye?

This study experimentally investigated whether the fluorescent intensity could be used as an index of the concentration of the fluorescent dye.

We prepared a fluorescent dye solution (CI-45350, Tokyo Chemical Industry, Tokyo, Japan) with a concentration of 1.06 mM, corresponding to the solution used in this study, as well as lower concentrations of 0.53, 0.33, 0.27, 0.21, 0.18, 0.15, and 0.13 mM . Figure S1.1 shows a schematic of the experimental setup. First, 50 μL of the solution was dropped into a large Petri dish (MS-11600, Sumitomo Bakelite, Tokyo, Japan), and then, a smaller Petri dish (MS-11350, Sumitomo Bakelite, Tokyo, Japan) was placed on the solution. These dishes were placed on the stage of a two-photon microscope (FV1200MPE, Olympus, Tokyo, Japan), and the fluorescent dye was observed using the G-channel bandpass filter (495–540 nm). An image stack with a size of 512 × 512 pixels (212 × 212 μm) in the x-y plane and 30 μm with every 5 μm in the z-direction (optical direction) was captured. The captured image was resliced from the y-direction, and a 2D x-z projection image was obtained as the maximum intensity. Then, the image in the x-direction was averaged, and the maximum value in the z-direction was defined as the intensity of the fluorescent solution.

Figure S1.2 shows the relationship between the fluorescent intensity and the concentration. The intensity is significantly and highly correlated with the concentration (R = 0.999, $p < 0.01$). This result indicates that the concentration can be evaluated from the intensity of the fluorescent image.

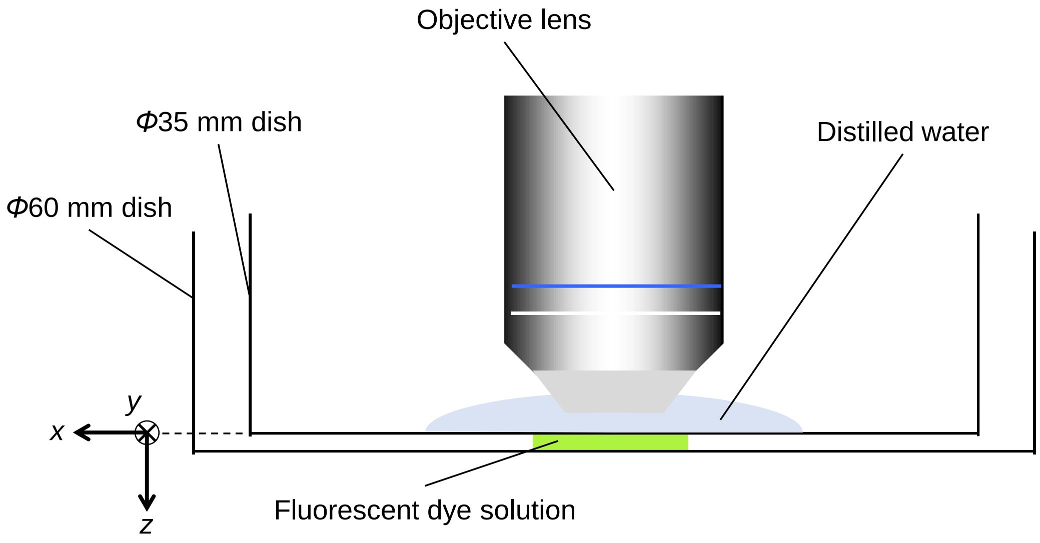

Fig. S1.1 A schematic illustration of intensity measurement of fluorescent dye.

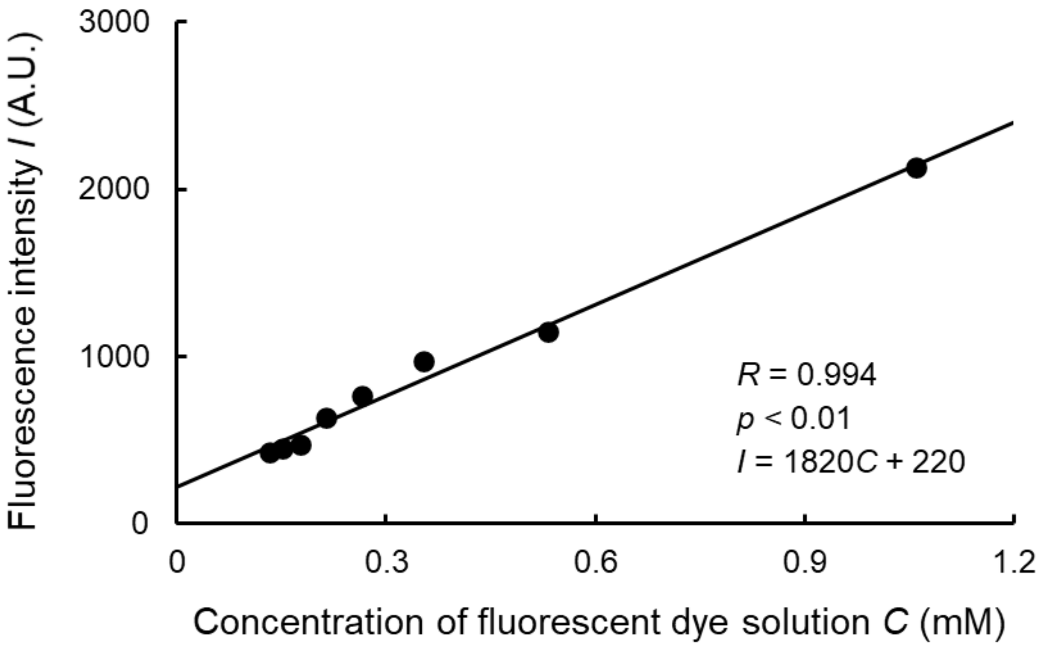

Fig. S1.2 Fluorescent intensity I plotted against the fluorescent dye concentration C. R, correlation coefficient.

S2. Result of kymograph, plots between *a'* and *b'*, and determined velocity *v* in ELs and SMLs

The kymograph, plots between *a'* and *b'*, and determined velocity *v* in each EL and SML as obtained for the interstitial flow velocity in the whole thickness of the aorta are shown in Figs. S2.1, S2.2, and S2.3, respectively. Because the time length of the kymographs was determined as the time at which the intensity almost reached the static state, the shorter kymograph means that the concentration has early reached the steady state and that the velocity (i.e., diffusion) is high there (Fig. S2.1). The plots between *a'* and *b'* show a good and high correlation (Fig. S2.2). In Fig. S2.3, negative values are often observed in ELs and even in SMLs (0–3 from 10–13 measurements). These results indicate the low accuracy of this measurement in ELs, and therefore, the EL data was not used in further analyses. The low accuracy in ELs was attributed to the nature of the fluorescent dye; specifically, the dye was bound more easily to the ELs than to SMLs (See Supplementary Materials S6). Under such conditions, the inverse intensity gradient, where the intensity on the adventitial side is higher than that on the intimal side in the *r*-direction, can be locally seen; it results in a negative slope in the plots between *a'* and *b'*.

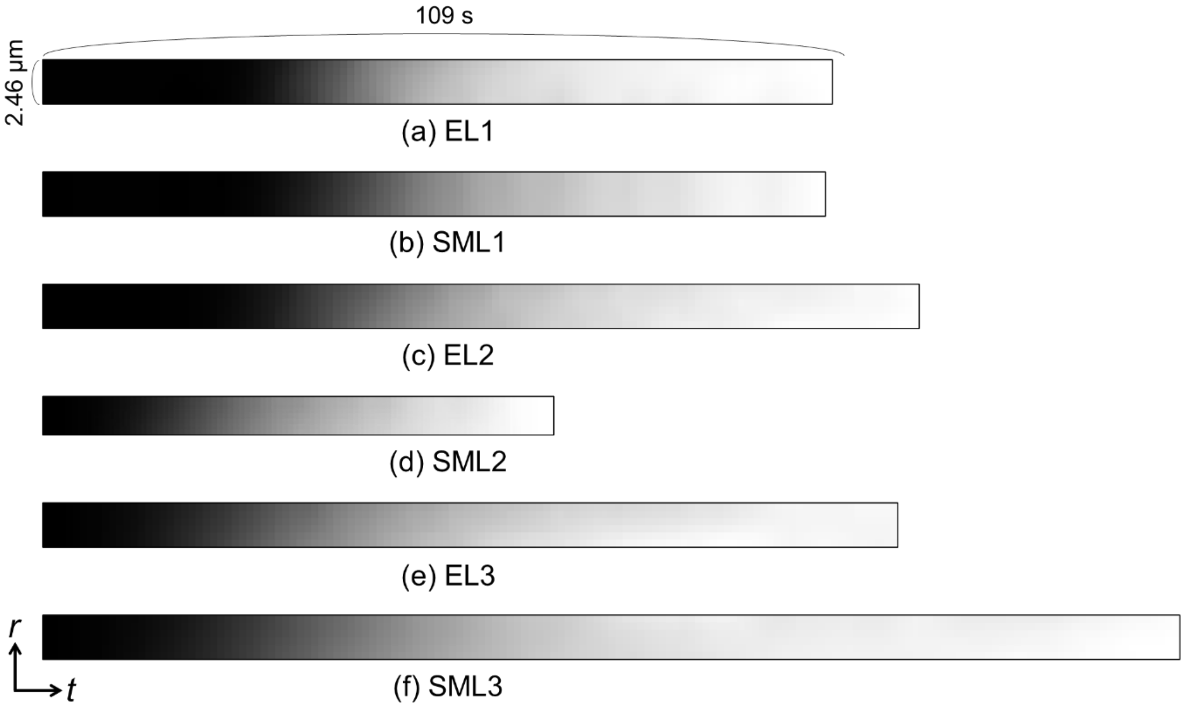

Fig. S2.1 Kymograph of fluorescent dye solution in (a) EL1, (b) SML1, (c) EL2, (d) SML2, (e) EL3, and (f) SML3 from the intraluminal side at intraluminal pressure of 160 mmHg. *t*, time axis; *r*, radial axis.

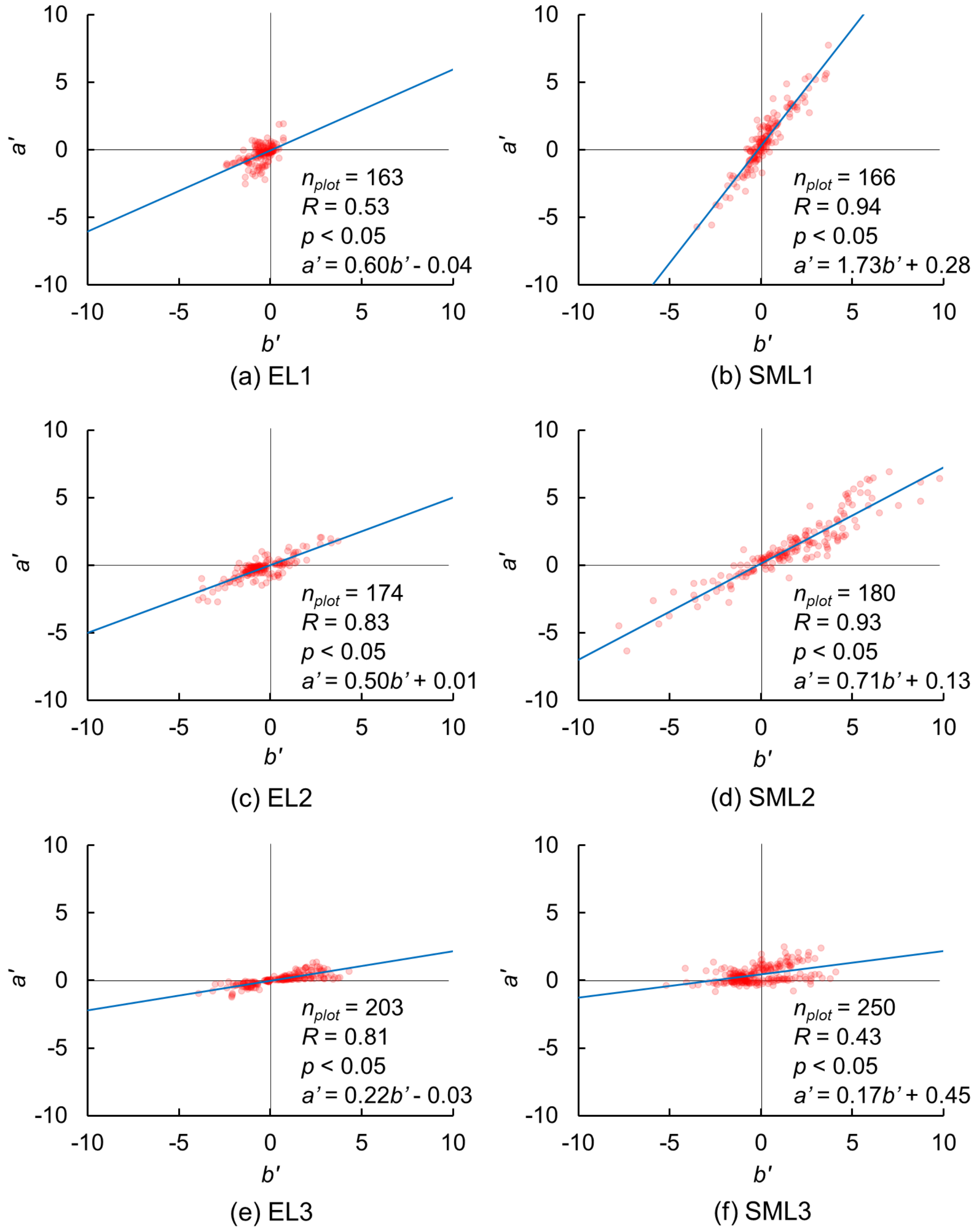

Fig. S2.2 Typical plots of a' and b' in Eq. (8) in (a) EL1, (b) SML1, (c) EL2, (d) SML2, (e) EL3, and (f) SML3 from the intraluminal side at intraluminal pressure of 160 mmHg. n_{plot}, number of plots.

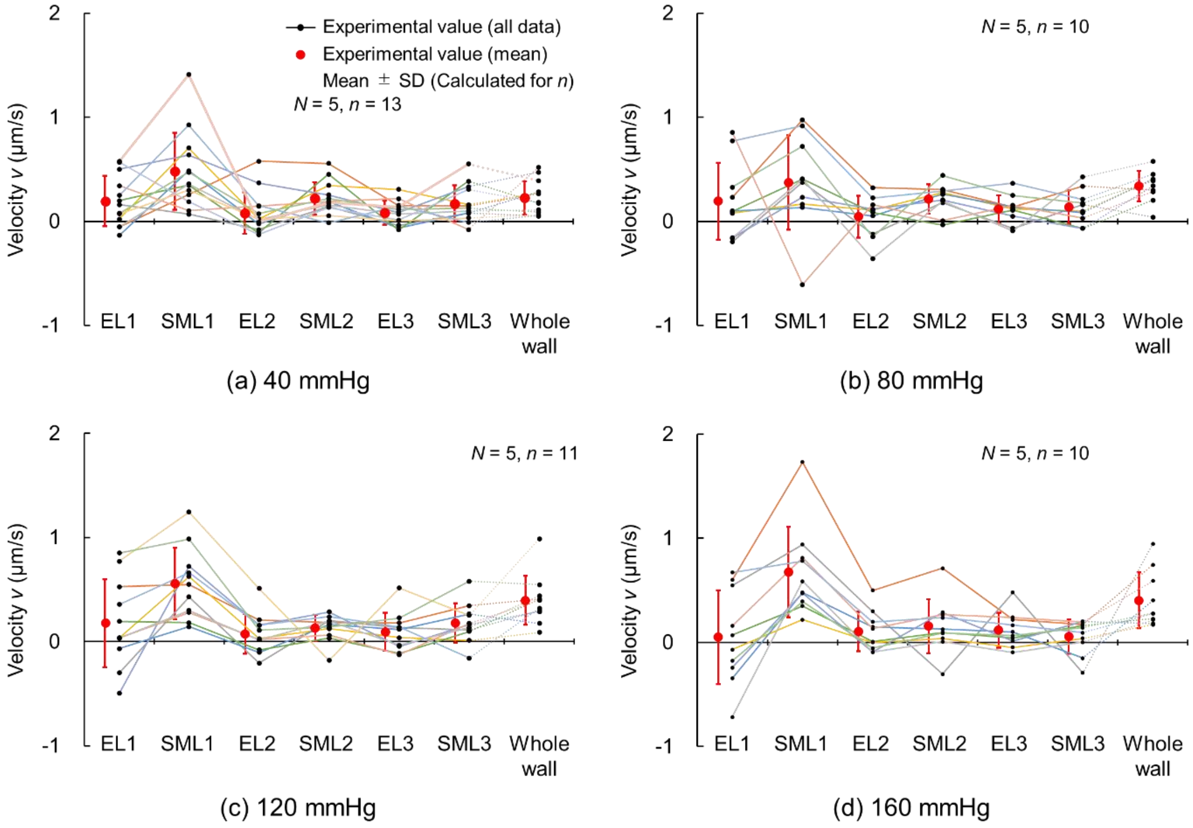

Fig. S2.3 Interstitial flow velocity in local ELs and SMLs along with the velocity of the whole wall at (a) 40, (b) 80, (c) 120, and (d) 160 mmHg. *N*, Number of mice; *n*, number of data.

S3. Effect of solution viscosity, EC existence, and experimental order on the interstitial velocity

To clarify the effect of solution viscosity and the existence of ECs on our interstitial velocity data, we performed additional experiments.

After the interstitial flow measurements were performed, the specimen was fixed with 10% buffered formalin, followed by the staining of VE-cadherin and nuclei with VE-cadherin antibody (sc-6458, Santa Cruz Biotechnology, Santa Cruz, CA, USA) and Hoechst 33342, respectively. The results are shown in Fig. S3.1. The ECs were recognized at the intraluminal surface with VE-cadherin staining. This figure also shows that ECs were partially detached from the intraluminal surface, and it was unclear whether EC existed in the region of our velocity measurement. We also found that when the EC nuclei were not stained with Hoechst 33342, and the ECs were detached. This observation indicates that ECs were recognized easily by nuclear staining with Hoechst 33342 without fixation and antibody staining. Thus, we measured the interstitial velocity at the position where the nuclei were clearly observed with Hoechst 33342. The 1.62 μM Hoechst 33342 solution was introduced in the intraluminal side of the aortic specimen and incubated for 30 min. Then, the solution viscosity was regulated to 1.2 mPa s in a Krebs–Henseleit buffer (2.3 mM $CaCl_2$, 115.3 mM NaCl, 4.6 mM KCl, 1.1 mM $MgSO_4$, 22.1 mM $NaHCO_3$, 1.1 mM KH_2PO_4, 7.8 mM glucose), and the interstitial velocity was measured. The result of the interstitial flow is shown in Fig. S3.2. The velocity measured at the first trial was 0.12 μm/s at 40 mmHg, 0.35 μm/s at 100 mmHg, and 0.38 μm/s at 160 mmHg. Thus, even if the ECs existed and solution viscosity was regulated, the interstitial velocity measured with our suggested method was higher than those in previous studies. The difference in interstitial velocities was not attributed to the existence of EC nor to the viscosity of the solutions.

In the experiment, the first velocity measurement was performed in the order of 40, 100, and 160 mmHg. Then, in the second measurement, the experimental sequence was reversed: 160, 100, and 40 mmHg. The result shows that at 100 mmHg, the first measured velocity was higher than the second measured velocity. However, at 160 mmHg, the first measured velocity was lower than the second measured velocity. Thus, the measurement

sequence does not influence the flow velocity in this study.

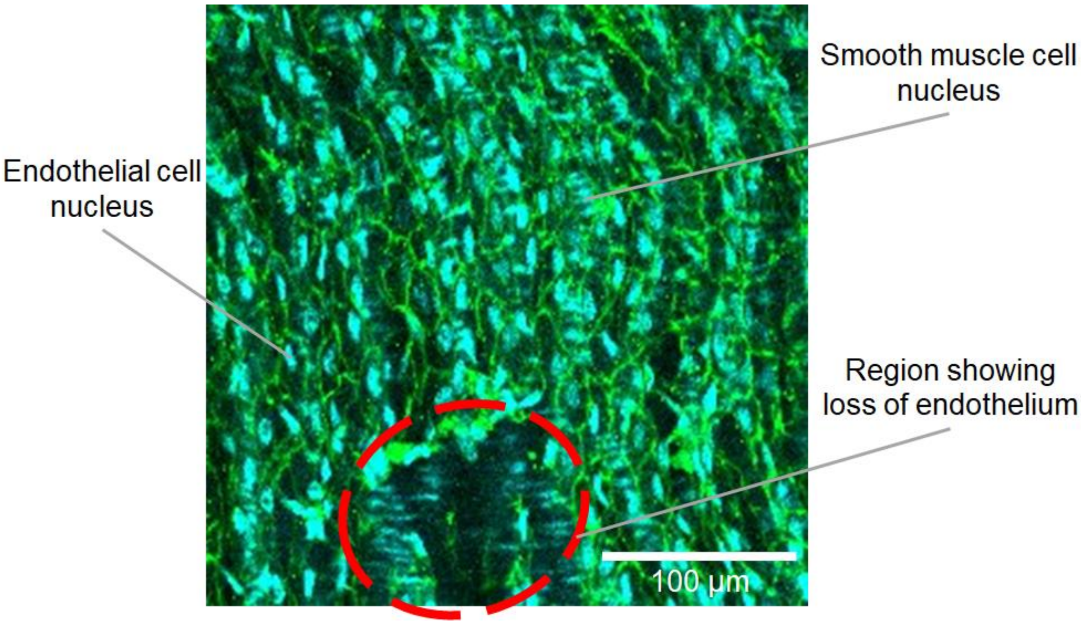

Fig. S3.1 Intraluminal surface of a mouse thoracic aorta stained with VE-cadherin (green) and Hoechst 33342 (cyan). The horizontal and vertical axes correspond to the circumferential and longitudinal axis, respectively.

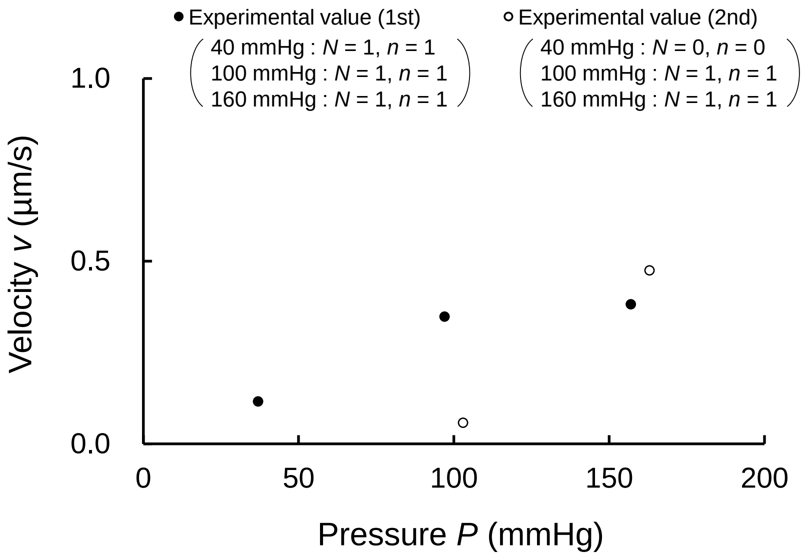

Fig. S3.2 Interstitial flow velocity measured at an EC existence region.

S4. Effect of adventitia on the fluorescent image in the aortic media.

Removal of adventitia is necessary to observe the fluorescent dye in the aortic media. Figure S4 shows the fluorescent image of the aorta in a border region with and without the adventitia. When the adventitia was not removed, observation in media was difficult.

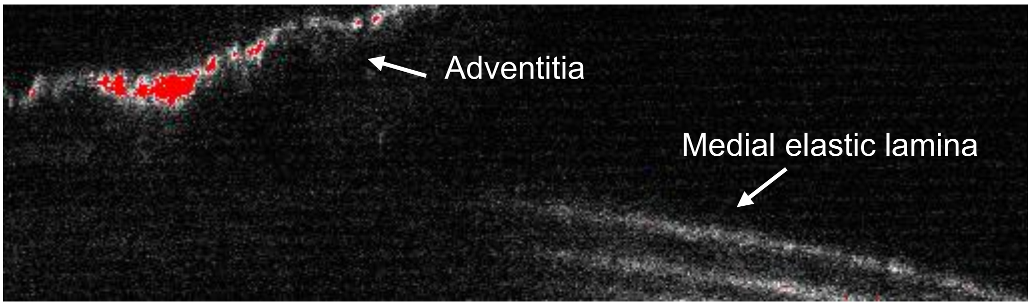

Fig. S4. Effect of adventitia on the fluorescent image in the aorta. Medial elastic laminas are observed in the region without adventitia (right side), while they are not observed in the region with adventitia (left side).

S5. Changes in wall thickness during pressurization

Because the aorta is viscoelastic, the wall thickness might decrease during pressurization, resulting in an increased pressure gradient through the aortic walls and interstitial flow velocity. Here, we investigated the wall thickness at the start time t_{start} and end time t_{end} of the captured image.

The images in Fig. 3 were used for this analysis. The distance between the internal and the external ELs (*i.e.*, r_{EEL}) was determined as the wall thickness W. The wall thickness was measured at t_{start} and t_{end}, and they were compared and tested with a paired Student's t-test for all pressure levels P. The correlation coefficient between W and P was tested using Student's t-test. The significant level was set as $p = 0.05$.

Figure S5.1 shows the wall thickness W at the start time t_{start} and end time t_{end}. W at t_{start} was comparable to W at t_{end} for all pressure levels, and there were no significant differences between them, indicating that the wall thickness did not change during the experiment. The wall thickness significantly and negatively correlated with the intraluminal pressure (R = -0.507 for t_{start}, $p < 0.05$; R = -0.505 for t_{end}, $p < 0.05$) as reported in Guo et al. [37]. Thus, the pressure gradient in the radial direction increased with an increase in the pressure owing to not only the pressure itself but also the reduction in the wall thickness.

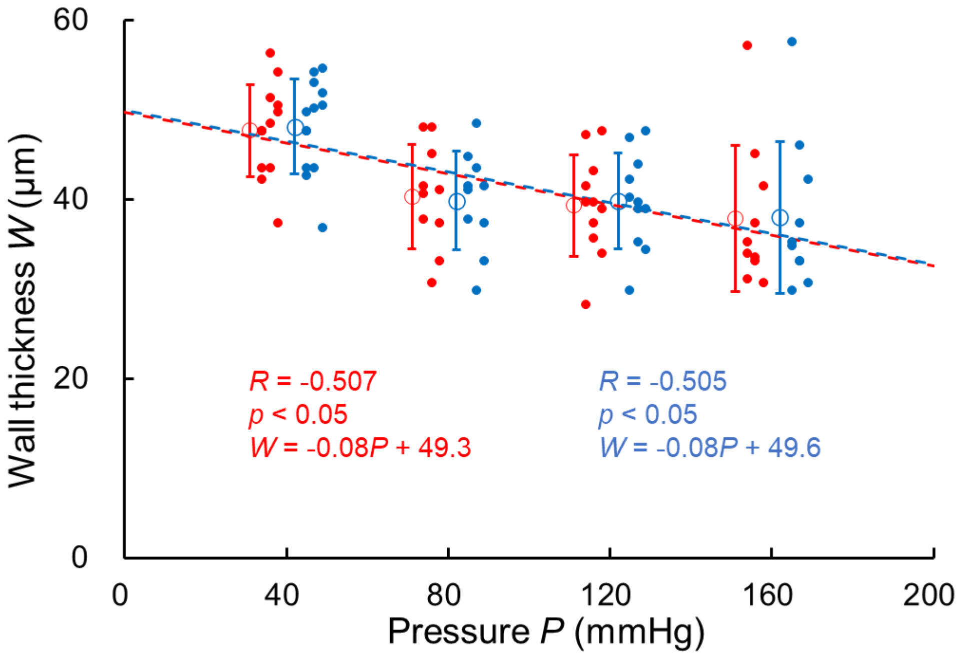

Fig. S5.1 Aortic wall thickness W plotted to intraluminal pressure P at the start time t_{start} (red) and end time t_{end} (blue). Number of mice $N = 5$; number of measurement data $n = 13$ at 40 mmHg, $n = 10$ at 80 mmHg, $n = 11$ at 120, and $n = 10$ at 160 mmHg; R, correlation coefficient.

S6. Why was the intensity in the EL region higher than that in the SML region?

The fluorescent intensity of the fluorescent dye solution was higher in ELs than in SMLs. This fluorescent intensity in ELs was not generated from the autofluorescence of elastin because the intensity was not high at time $t = t_{\mathrm{start}}$ (images in top row in Fig. 4). Thus, we hypothesized that the fluorescent dye, uranine, is more bound to ELs than to SMLs, and we evaluated this hypothesis.

Aortic samples were sliced with a microslicer as described in a previous report [38]. The samples were immersed in 1.06 mM uranine fluorescent dye solution for 5 min at room temperature and then washed with the buffer. A sample image was observed under the two-photon microscope. A 512 × 512 pixel (212 μm × 212 μm) image stack (2.00-μm intervals between image sectioning and total thickness of 40–80 μm) was captured. After taking the maximum intensity projection, the intensities in both regions were measured by tracing the regions of ELs and SMLs using ImageJ image analysis software.

Figure S6.1 shows the fluorescent images before and after immersing the specimen in the fluorescent dye solution. The intensity after immersion is clearly higher in the ELs than in SMLs. Figure S6.2 shows the quantified intensity in ELs and SMLs after immersion in the fluorescent dye solution. In this graph, the intensity is normalized with the intensity before immersion in the fluorescent dye solution to remove the effect of autofluorescence. The fluorescent intensity in ELs is significantly higher than that in SMLs. Thus, we conclude that the uranine fluorescent dye binds more to ELs than to SMLs.

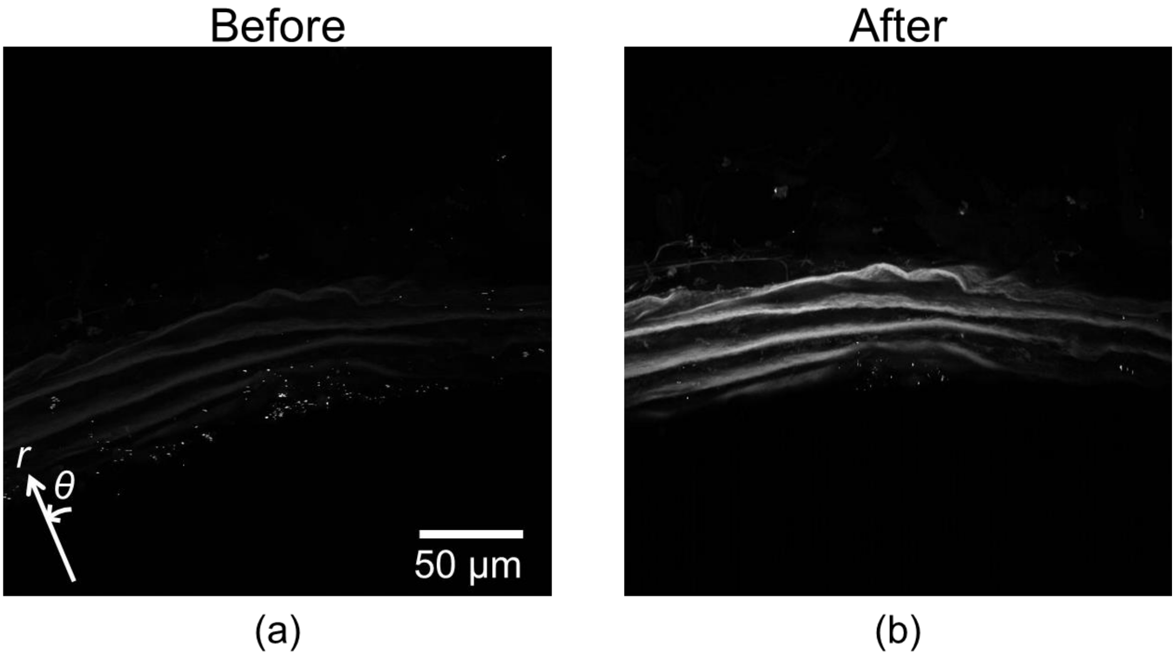

Fig. S6.1 Fluorescent images of the aorta (a) before and (b) after immersion in the fluorescent dye solution. In (b), the fluorescent intensity in ELs is much higher than that in SMLs.

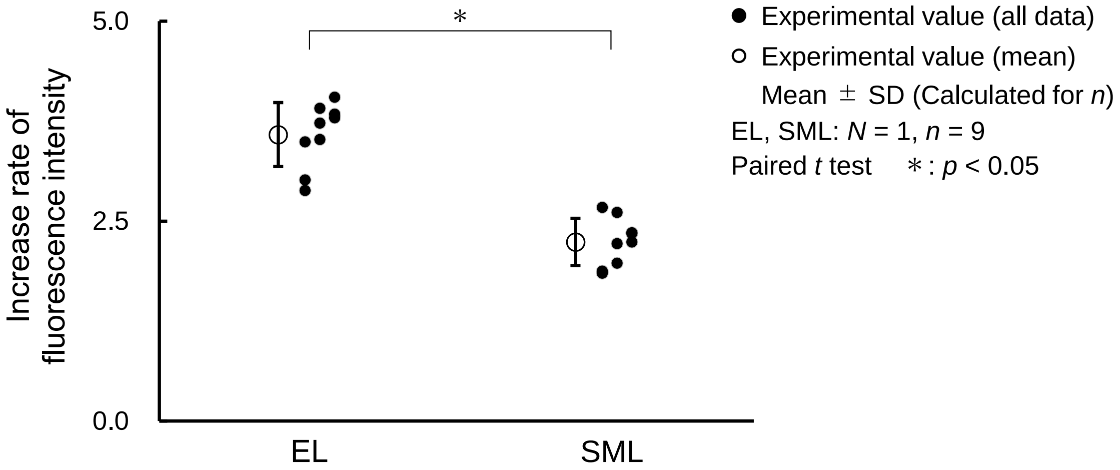

Fig. S6.2 The ratio of the fluorescent intensity before immersion to that after immersion in the fluorescent dye solution in ELs and SMLs. N, Number of mice; n, number of data.

S7. Comparison of convective velocity with diffusive velocity

Because the fluorescent dye moves by both the interstitial flow and diffusion, we compared the magnitudes of both effects. To evaluate the magnitudes of the velocity and diffusion, the Peclet number *Pe*, which shows the ratio of convective velocity to diffusive velocity, was calculated according to Baldwin et al. [19] as follows:

$$Pe = \frac{v}{P_m}(1 - \sigma_f), \qquad \text{(S7.1)}$$

where P_m is the permeability coefficient and σ_f, the filtration reflection coefficient. $Pe > 1$ indicates that the convective velocity dominates over the diffusive velocity. The diffusion coefficient k is obtained as the division of P_m and the wall thickness W:

$$Pe = \frac{vW}{k}(1 - \sigma_f). \qquad \text{(S7.2)}$$

Shou et al. [4] estimated σ_f of trypan blue (molecular weight: 961) as 0.064. Because the molecular weight of uranine is 376, σ_f of uranine must be <0.064. The averaged v and k values obtained in this study are 0.33 μm/s and 0.38 μm^2/s (40–160 mmHg), respectively, and the averaged thickness is $W = 41.8$ μm. By using $\sigma_f = 0.064$, Pe is calculated as 34. Because σ_f of uranine must be <0.064, the Peclet number is >34. This indicates that the diffusion velocity in the aorta is much smaller than the convective velocity.

S8. Measurement of diffusion coefficient without flow

The proposed method can measure the diffusion coefficient as well as the flow velocity. Because the movement of the fluorescent dye by diffusion was much smaller than that by flow (see Supplementary Materials S7), we did not have confidence in the accuracy of the measured diffusion coefficient. Thus, to verify the magnitude of the diffusion coefficient, we measured the diffusion coefficient under the condition without flow.

We tried to measure only the diffusion coefficient. However, under zero intraluminal pressurization, the shape of the aorta cannot be maintained as a cylindrical pipe, resulting in a difficulty in observing the fluorescent dye. Thus, we applied an intraluminal pressure of only 5 mmHg to maintain the shape of the aortic sample and a small interstitial flow in the aorta in the experiments described in this section.

The three-way valve (no. 3 in Fig. 1) was opened, and the intraluminal pressure was kept at 80 mmHg until the fluorescent dye solution reached the aortic sample. Then, the pressure applied by the electropneumatic regulator was temporarily stopped and a hydrostatic pressure of 5 mmHg was applied to the intraluminal side of the aorta by vertically elevating the reservoir. The fluorescent dye was observed and image analysis was performed.

Figure S8.1a shows a time-lapsed image of fluorescent in the aortic wall at 5 mmHg in the radial-circumferential (r–θ) plane. The fluorescent intensity gradually increased with increasing time, indicating that the fluorescent dye moved into the aortic walls. After 200 s, the intensity in the adventitial side of the media became 0, indicating that this side was locally destroyed by laser ablation. Because the adventitial side of the media was destroyed, only the intimal side (region within 29 μm from the internal EL) was analyzed. Further, only images captured until 200 s were used because the intensity in the intimal side suddenly increased. This might have been caused by the destruction of the adventitial side, and the laser easily reached the intimal side. The average intensity in the analyzed region until 200 s (Fig. S8b) and the normalized kymograph (Fig. S8c) clearly show the increase in the fluorescent intensity with increasing time. Figure S8d shows the plots of (a', b'). The fitted linear regression line was $a' = 0.01b' + 0.16$, and no significant correlation was observed. This result confirms that the interstitial flow was negligibly small (0.01 μm/s) at 5 mmHg. The diffusion coefficient (k

= 0.16 μm^2/s) almost concurred with the data at 40 mmHg (k = 0.24 ± 0.18 μm^2/s). Therefore, we confirmed that the diffusion coefficients in the aortic media as measured using the proposed method were appropriate even under higher intraluminal pressures.

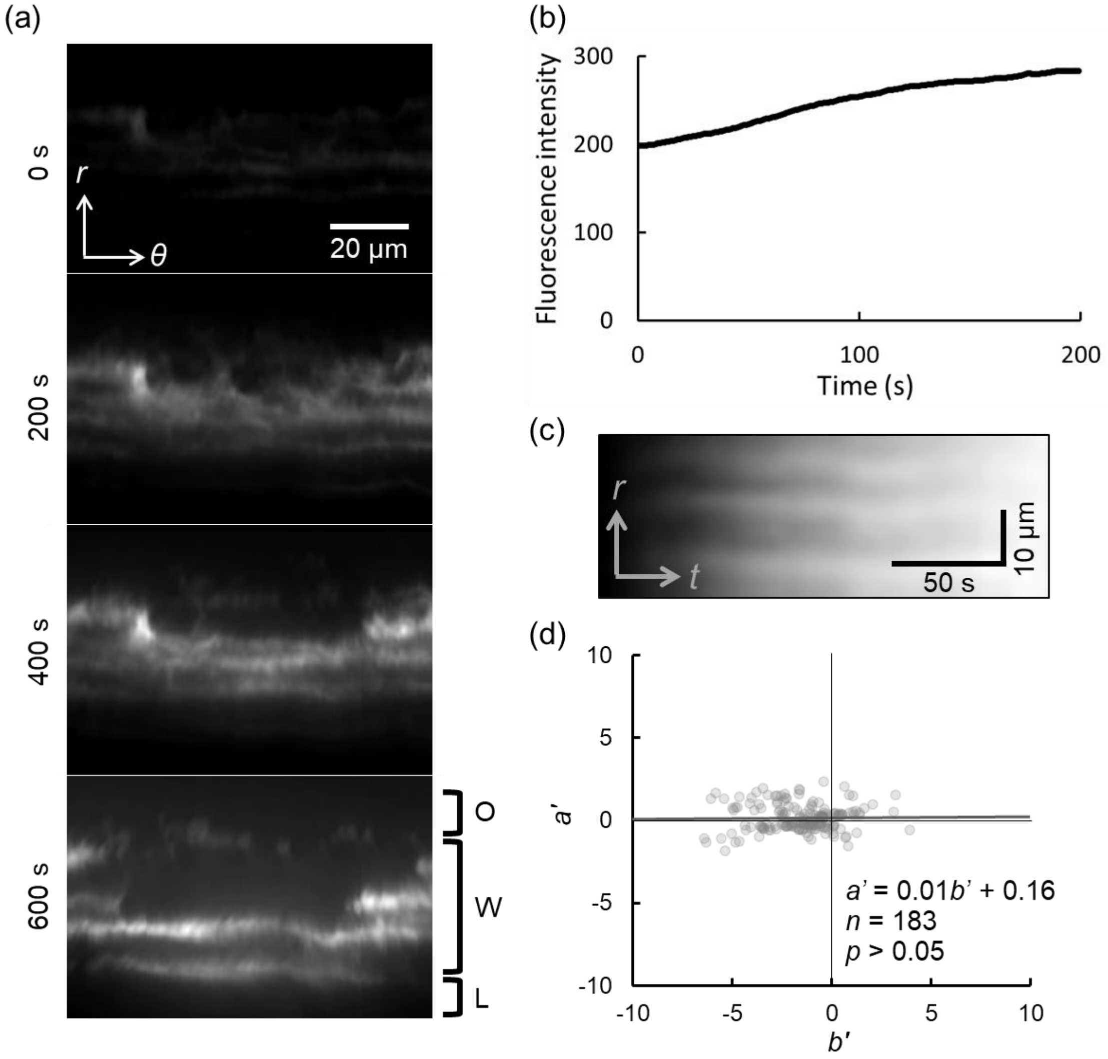

Fig. S8.1 Images of interstitial flow in the aortic wall at intraluminal pressure of 5 mmHg, and image analysis processes of interstitial flow velocity and diffusion coefficient. (a) Time-lapsed images of the aortic walls in the radius-circumferential (*r*-*θ*) cross-section at intraluminal pressure of 5 mmHg. Images were captured at 0, 200, 400, and 600 s after capturing a photograph. From the top to the bottom sides, the outside of the aorta (O), aortic wall (W), and lumen (L) are shown. (b) Time-course change in the average fluorescence intensity taken on the intimal side of the aortic wall. (c) A normalized kymograph with 0 at its left edge and 1 at its right edge. The normalization was performed at each *r*-axis. *t*, time axis. (d) Plots of *a*' and *b*' at intraluminal pressure of 5 mmHg. *n*, number of plots.

Movie 1 Time-lapsed images of ELs (red) and fluorescent dye (green) solutions in the r-θ plane at 40, 80, 120, and 160 mmHg.
